# Treatment of transthyretin (TTR) amyloid cardiomyopathy with an antisense oligonucleotide inhibitor of TTR synthesis

**DOI:** 10.1186/1750-1172-10-S1-P7

**Published:** 2015-11-02

**Authors:** Merrill D Benson, Elizabeth J Ackermann, Brett P Monia

**Affiliations:** 1 Indiana University School of Medicine, Pathology and Laboratory Medicine, 46202, Indianapolis, USA; 2 ISIS Pharmaceuticals, Inc., Management, 92010, Carlsbad, USA

## Background

While transthyretin (TTR) amyloidosis is usually characterized by peripheral and autonomic neuropathy, a majority of patients also have evidence of restrictive cardiomyopathy. In addition, amyloid cardiomyopathy may occur in the elderly without the presence of a mutant form of TTR. Both the hereditary and wild-type TTR amyloidosis are characterized by progressive restrictive cardiomyopathy. The disease usually progresses over a five to ten year period from time of diagnosis to demise from congestive heart failure.

At present there are ongoing pharmaceutical studies to suppress the synthesis of TTR by the liver using antisense oligonucleotides or siRNA. Both types of agents have been shown to be effective in lowering blood levels of TTR but efficacy measured by inhibition of progression of disease has not yet been established. The present study is an investigator sponsored Phase-2 study to determine the safety and tolerability of an ISIS generation 2.0 antisense oligonucleotide in patients with moderate to advanced TTR cardiomyopathy.

## Methods

Subjects are admitted to the study with biopsy proven transthyretin amyloidosis (ATTR) and a left ventricular (LV) wall thickness of 1.3 cm. Safety parameters are observed over a 24-month period. Echocardiography is conducted at baseline and at 6-month intervals. Cardiac MRI is obtained at baseline and 12-month intervals. ISIS-TTRRx 300 mg is administered weekly by subcutaneous injection.

## Results

To date, ten patients have been admitted to the study. Five patients have completed greater than 6-months on drug. TTR plasma levels have fallen steadily with reduction up to 88% and a mean suppression of 78% by 39-weeks. No serious adverse events have occurred.

## Conclusion

ISIS-TTRRx appears to be well tolerated by patients with ATTR cardiomyopathy. Efficacy evaluation awaits longitudinal studies determining stability of LV mass.

